# In vivo efficacy of anti-malarial drugs against clinical *Plasmodium vivax* malaria in Ethiopia: a systematic review and meta-analysis

**DOI:** 10.1186/s12936-021-04016-2

**Published:** 2021-12-24

**Authors:** Tsige Ketema, Ketema Bacha, Kefelegn Getahun, Quique Bassat

**Affiliations:** 1 Department of Biology, College of Natural Sciences, Jimma University, Jimma, Ethiopia; 2 ISGlobal, Hospital Clínic - Universitat de Barcelona, Barcelona, Spain; 3 Department of Geography and Environmental Studies, College of Social Sciences and Humanity, Jimma University, Jimma, Ethiopia; 4 Catalan Institution for Research and Advanced Studies, ICREA, Pg. Lluís Companys 23, 08010 Barcelona, Spain; 5 Centro de Investigação Em Saúde de Manhiça (CISM), Maputo, Mozambique; 6 Pediatrics Department, Hospital Sant Joan de Déu, Universitat de Barcelona, Esplugues, Barcelona, Spain; 7 Consorcio de Investigación Biomédica en Red de Epidemiología Y Salud Pública (CIBERESP), Madrid, Spain

**Keywords:** Anti-malarial drug, Artemether-lumefantrine, Chloroquine, Ethiopia, Efficacy, In vivo, Primaquine, Plasmodium vivax

## Abstract

**Background:**

Ethiopia is one of the few countries in Africa where *Plasmodium vivax* commonly co-exists with *Plasmodium falciparum*, and which accounts for ~ 40% of the total number of malaria infections in the country. Regardless of the growing evidence over many decades of decreasing sensitivity of this parasite to different anti-malarial drugs, there has been no comprehensive attempt made to systematically review and meta-analyse the efficacy of different anti-malarial drugs against *P. vivax* in the country. However, outlining the efficacy of available anti-malarial drugs against this parasite is essential to guide recommendations for the optimal therapeutic strategy to use in clinical practice. The aim of this study was to synthesize evidence on the efficacy of anti-malarial drugs against clinical P. vivax malaria in Ethiopia.

**Methods:**

All potentially relevant, peer-reviewed articles accessible in PubMed, Scopus, Web of Science, and Clinical Trial.gov electronic databases were retrieved using a search strategy combining keywords and related database-specific subject terms. Randomized controlled trials (RCTs) and non-randomized trials aiming to investigate the efficacy of anti-malarial drugs against *P. vivax* were included in the review. Data were analysed using Review Manager Software. Cochrane Q (χ^2^) and the *I*^2^ tests were used to assess heterogeneity. The funnel plot and Egger’s test were used to examine risk of publication bias.

**Results:**

Out of 1294 identified citations, 14 articles that presented data on 29 treatment options were included in the analysis. These studies enrolled 2144 clinical vivax malaria patients. The pooled estimate of in vivo efficacy of anti-malarial drugs against vivax malaria in Ethiopia was 97.91% (95% CI: 97.29–98.52%), with significant heterogeneity (*I*^2^ = 86%, p < 0.0001) and publication bias (Egger’s test = -12.86, p < 0.001). Different anti-malarial drugs showed varied efficacies against vivax malaria. The duration of follow-up significantly affected the calculated efficacy of any given anti-malarial drug, with longer duration of the follow-up (42 days) associated with significantly lower efficacy than efficacy reported on day 28. Also, pooled PCR-corrected efficacy and efficacy estimated from altitudinally lower transmission settings were significantly higher than PCR-uncorrected efficacy that estimated for moderate transmission settings, respectively.

**Conclusion:**

The overall efficacy of anti-malarial drugs evaluated for the treatment of vivax malaria in Ethiopia was generally high, although there was wide-ranging degree of efficacy, which was affected by the treatment options, duration of follow-up, transmission intensity, and the confirmation procedures for recurrent parasitaemia. Regardless of evidence of sporadic efficacy reduction reported in the country, chloroquine (CQ), the first-line regimen in Ethiopia, remained highly efficacious, supporting its continuous utilization for confirmed *P. vivax* mono-infections. The addition of primaquine (PQ) to CQ is recommended, as this is the only approved way to provide radical cure, and thus ensure sustained efficacy and longer protection against *P. vivax*. Continuous surveillance of the efficacy of anti-malarial drugs and clinical trials to allow robust conclusions remains necessary to proactively act against possible emergence and spread of drug-resistant *P. vivax* in Ethiopia.

**Supplementary Information:**

The online version contains supplementary material available at 10.1186/s12936-021-04016-2.

## Background

*Plasmodium vivax* is the most widespread malaria parasite species, and it infects around 14 million people globally every year [1]. Most of these cases are reported from the Asia–Pacific Region, Central and South America, the Middle East, Oceania and East Africa [2, 3]. Before the contradictory reports on vivax infection of Duffy antigen-negative populations, in West-Central Africa and Madagascar among Malagasy people, appeared [4, 5], *P. vivax* was considered a species that seldom circulated in sub-Saharan Africa. In Ethiopia, and some East African countries, it is a clear source of malaria infections and clinical disease [6]. During the past few years, the global malaria burden has been steadily decreasing, but the last years have seen a stagnation of progress [7]. The remarkable improvements witnessed in the first 15 years of the millennium have been achieved largely because of strong commitments of governments and concerned bodies in malaria-endemic areas, sustainable support from partner organizations, availability of relatively better diagnostic options, and extensive utilization of, as well as accessibility to, different interventional tools [8]. However, this multidimensional effort has been compromised by the emergence of drug-resistant *Plasmodium* parasites in most malaria-endemic regions of the world, together with various other biological challenges, which threaten further progress.

Regardless of the growing evidence for the decreasing efficacy of chloroquine (CQ) against *P. vivax* in Ethiopia during the last two decades [9], CQ remains the first-line drug for treatment of P. vivax malaria [10]. In some other *P. vivax-*endemic countries however, this drug is no longer in use as CQ-resistant *P. vivax (*CR*Pv)* parasites have emerged and become widely disseminated [11], or because of the convenience of having a single first-line treatment in place (normally based on artemisinin-combination therapy), irrespective of the infecting species. The recurrent episodes due to drug-resistant *P. vivax* could increase vulnerability to other health problems and ultimately lead to severe outcomes [12]. In addition, CQ does not provide a radical cure for P. vivax malaria, therefore requiring its supplementation with a drug active against the parasite’s dormant liver stages (hypnozoites) [13], such as primaquine (PQ) or more recently tafenoquine (TQ), although they are schizonitcidal, too [14, 15]. Since PQ and TQ are 8‐aminoquinoline anti-malarial drugs that can cause severe haemolysis in individuals with glucose-6-phosphate dehydrogenase (G6PD) enzyme deficiency, their use for radical cure should always be accompanied by prior checking of the status of the enzymatic activity [16, 17]. Hypnozoites, which can apparently result in multiple malaria episodes following even a single mosquito bite, and together with persisting stages, such as bone marrow [18] or spleen [19], are serious challenges to efforts being made to eliminate and eradicate malaria globally [20, 21].

Many studies from Ethiopia have reported a decreasing sensitivity of *P. vivax* to CQ [22–24], although this appears sporadic as some studies showed sustained efficacy of this drug [25–27]. Although not officially recommended in the Ethiopian malaria treatment guidelines, studies have investigated the efficacy of alternative treatments for vivax malaria, such as treatment with artemether-lumefantrine (AL); CQ combined with PQ; or, AL with PQ [25, 28–30]. The aim of the present study was to systematically review existing evidences concerning the efficacy of different anti-malarial drugs against clinical vivax malaria in Ethiopia, and to synthesize available data in order to outline its pooled efficacy. This is to better guide future recommendations for anti-malarial policy in Ethiopia.

## Methods

### Research design

The study was conducted in accordance with Preferred Reposting Items for Systematic Reviews and Meta-Analyses (PRISMA) guidelines. The protocol for this review was registered at PROSPERO International Prospective Register of Systematic Reviews, with ID: CRD42020201761 [31].

### Data source and search strategies

**Fig. 1:**
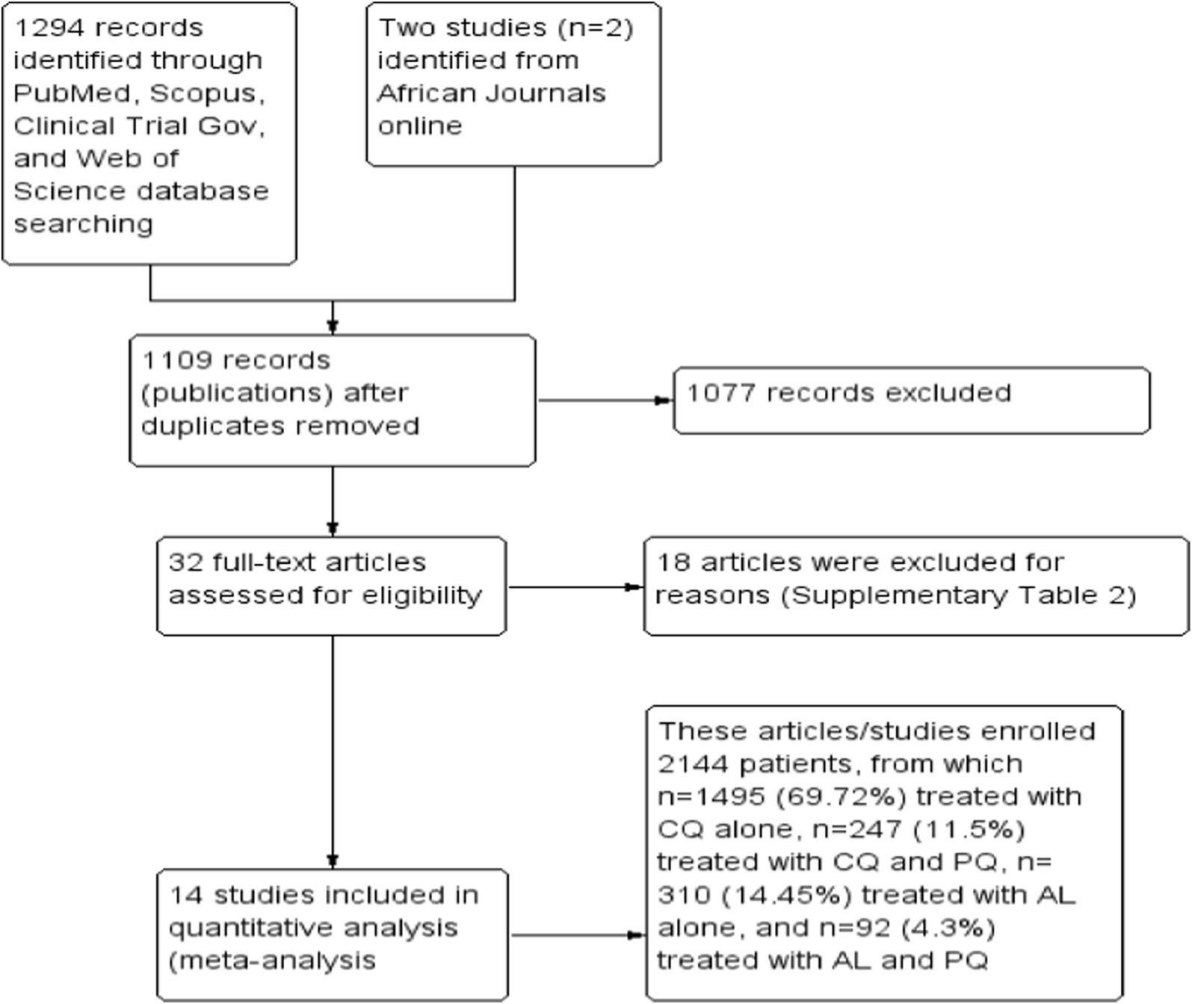
Study flow diagram

Related articles were gathered from the major electronic databases: PubMed (n = 1057), Web of Science (n = 87), Scopus (n = 132), and Clinical Trial.gov (n = 18) (Fig. 1). The search strategy for each database was developed using MeSH and free-text words to capture articles addressing in vivo efficacy of anti-malarial drugs against clinical vivax malaria in Ethiopian populations, without language restrictions (Additional file 3: Table S1). The search strategy was applied to articles published since the year 2000. The last search was performed on 31 March 2021. In addition, an effort was made to retrieve more information manually from regional and local journals such as *African Journal Online* (AJOL) (n = 2). Grey literature and non-published data were not included in the review. Results from different database searches were aggregated and any duplicated data/studies were removed.

### Eligibility of the studies

One of the criteria used to check eligibility for inclusion was originality of publications describing in vivo efficacy of anti-malarial drugs against *P. vivax* in Ethiopian populations. Furthermore, clinical trials, randomized open-labelled, randomized controlled, and single arm open-label, written in any language and published from 1 January, 2000 to 31 March, 2021 were included. Other publication types such as reviews, conference abstracts, commentaries, editorials, registered protocols for clinical trials, letters to the editor, personal opinions, non-human or in vitro or in vivo studies in animals, studies on other *Plasmodium* species, and those without clinical trial or interventional studies were excluded.

### Study selection

Two authors (TK and KB) independently screened titles and abstracts of all records identified by the search strategy for potential inclusion in the review. Thereafter, full-text copies of articles deemed potentially relevant were retrieved and their eligibility was assessed. Disagreements between individual judgements were resolved through discussion. All excluded studies were listed and reasons given for their exclusion (Additional file 3: Table S2). Key characteristics of included studies were extracted using a format prepared in accordance with the *PICOS model for clinical questions* [32] (Table 1).

**Table 1:** PICOS strategies

PICOS	Characteristic criteria for inclusion
P: population	The study population were *P. vivax* mono-infected clinical malaria patients (all age groups) seeking medication at health facilities in Ethiopia, who fulfilled the inclusion criteria set by WHO for anti-malarial drug efficacy testing
I: Intervention/exposure	Studies included in the current review followed any one or more of the following intervention strategies: fixed dose of CQ given for 3 consecutive days (2:2:1 ratio each day with a target total dose of 25 mg/kg, alone or combined with 0.25 mg/kg of PQ for 14 days); or AL (20 mg of artemether and 120 mg of lumefantrine based on body weight, alone or combined with 0.25 mg/kg of PQ for 14 days); all anti-malarial drugs were orally administered (fully or partially supervised), and patients followed for a minimum of 28 days
C: comparison/ control	Any placebo or anti-malarial drugs other than CQ, such as PQ and AL or different combination treatments
O: outcomes	Primary outcomes: parasitological and clinical efficacy of anti-malarial drugs, PCR-corrected or uncorrected late parasite recurrence or plasma drug level measured Major treatment outcomes [33] were: **Treatment failure (TF): Early treatment failure (ETF)**: any danger signs or severe malaria on days 1, 2 or 3 in the presence of parasitaemia; or parasitaemia on day 2 higher than on day 0, irrespective of axillary temperature; or parasitaemia on day 3 with axillary temperature ≥ 37.5ºC; or parasitaemia on day 3 ≥ 25% of count on day 0 **Late clinical failure (LCF):** danger signs or severe malaria in the presence of parasitaemia on any day between days 4 and 28 or 42 in patients who did not previously meet any of the criteria of ETF; or presence of parasitaemia on any day between days 4 and 28 or 42 with axillary temperature ≥ 37.5ºC; or history of fever in patients who did not previously meet any of the criteria of ETF **Late parasitological failure (LPF):** presence of parasitaemia on any day between days 7 and 28 or 42 with axillary temperature < 37.5ºC in patients who did not previously meet any of the criteria of ETF or LCF **Adequate clinical and parasitological response (ACPR):** if there was no parasitaemia on the follow-up days (28 or 42) irrespective of axillary temperature in patients without ETF, LCF or LPF. This is considered treatment success In addition, if the level of drug (CQ-DCQ) on day of recurrence is ≥ 100 ηg/ml (above minimum effective concentration (MEC)), the reappeared parasites were considered resistant to CQ, irrespective of genotype (relapse, recrudescence or re-infection) and classified as CQ-resistant *P. vivax* [34]
S: Studies	a.Randomized controlled trials (RCTs), non-randomized single-arm interventional studies (with or without a control group) and prospective cohort studies which enrolled all age groups, symptomatic patients with confirmed diagnosis of *P. vivax* mono-infection malaria, and who were followed-up for at least 28 days post-treatment b.Studies that assessed the efficacy of a fixed dose of CQ as a single arm, or randomized into different loose combinations of CQ plus PQ, and AL plus PQ

### Data extraction and management

Using a form, the two authors (TK and KB) independently extracted data on study characteristics such as author’s names, study site/region and study period, methodological characteristics (study design, sample size (number enrolled, and those who completed the follow-up)), treatment options (CQ alone OR combined with PQ (CQ plus PQ), AL alone OR combined with PQ (AL plus PQ)), and doses, follow-up days (28 or 42), gender, age, and outcome characteristics (TF, ETF, LTF, ACPR), those excluded/withdrawal, and re-infection with *Plasmodium falciparum*/mixed infection, efficacy of fever and parasite clearance, and confirmatory molecular tests for classification of recurrent parasitaemia into resumed relapse, recrudescent or new infection, although it was challenging (PCR corrected/PCR-uncorrected), and malaria transmission stratification (low (1751 and 2000 m), moderate (1001 and 1750 m), and high (< 1000 m)) as per 2021 mapping by the Ministry of Health of Ethiopia [35].

### Assessment of risk of bias in the included studies

The risk of bias for each included study was assessed independently using the Cochrane Handbook for Systematic Reviews of Interventions [36]. The critical appraisal tools are meant to assess the quality of studies reporting in vivo efficacy of anti-malarial drugs against vivax malaria in Ethiopia using seven critical appraisal domains: random sequence generation (selection biases), allocation concealment (selection bias), blinding of participants and personnel (performance bias), blinding of outcome assessment (detection bias), incomplete outcome date (attrition bias), reporting bias and other biases. An overall risk of bias was determined for each study, which was subsequently classified as low, unclear or high [36] (Additional file 1: Fig. S1a and b, and Additional file 3: Table S3).

### Data synthesis and analysis

Data were analysed using the Cochrane Review Manager (version 5.4) for qualitative and quantitative synthesis. Pooled, estimated treatment efficacy for each study was reported. Standard error of the mean (SE) for each study was calculated from the standard deviation obtained using the formula, *StDev* = $\sqrt{p (1-p)},$ where **p** is a proportion of the population with the treatment success. Then, SE was calculated from the *StDev* using the formula, *SE* = *StDev* $\sqrt{n}$, where n is the sample size (those who completed the follow-up).

Heterogeneity between studies was assessed using Cochrane’s Q (χ^2^) and the *I*^*2*^ tests. For the Cochrane’s test, a p-value of the χ^2^ test less than 0.05 was considered as significant statistical heterogeneity. *I*^*2*^ values of 25%, 50% and 75% were considered to represent low, medium and high heterogeneity, respectively. Due to considerable heterogeneity (*I*^*2*^ > 75%, p < 0.05), a random effects model was used to obtain the pooled, estimated in vivo efficacy of anti-malarial drugs against clinical vivax malaria.

Sub-group analysis was conducted to investigate heterogeneity. Pre-specified sub-groups potentially expected to affect the overall in vivo efficacy estimate included: treatment options (CQ alone OR in combination with PQ (CQ plus PQ), OR AL alone or in combination of PQ), follow-up durations (28 or 42 days), and confirmatory tests for recurrent parasitaemia (PCR-corrected and PCR-uncorrected). Forest plots were used to display point estimates and confidence intervals. Publication bias for studies included in the meta-analysis was assessed quantitatively using the Egger’s test and qualitatively by constructing a funnel plot and looking for asymmetry. ArcGIS software version 10.0 was used to sketch a map showing districts/regions from where anti-malarial drug efficacy estimates were reported.

## Results

### Study selection

A total of 1296 citations/records were initially identified. After the duplicates were excluded, 1109 unique citations were screened and assessed for eligibility. From the remaining 1109 screened at title/abstract level, a total of 1077 records considered irrelevant for the purposes of the study were excluded. At the second phase of record assessment, a total of 32 eligible studies with available full text were carefully reviewed and 14 articles were included for qualitative and quantitative meta-analysis (Fig. 1). Detailed reasons for excluding the other 18 studies are presented in Additional file 3: Table S2.

### Study characteristics

**Fig. 2:**
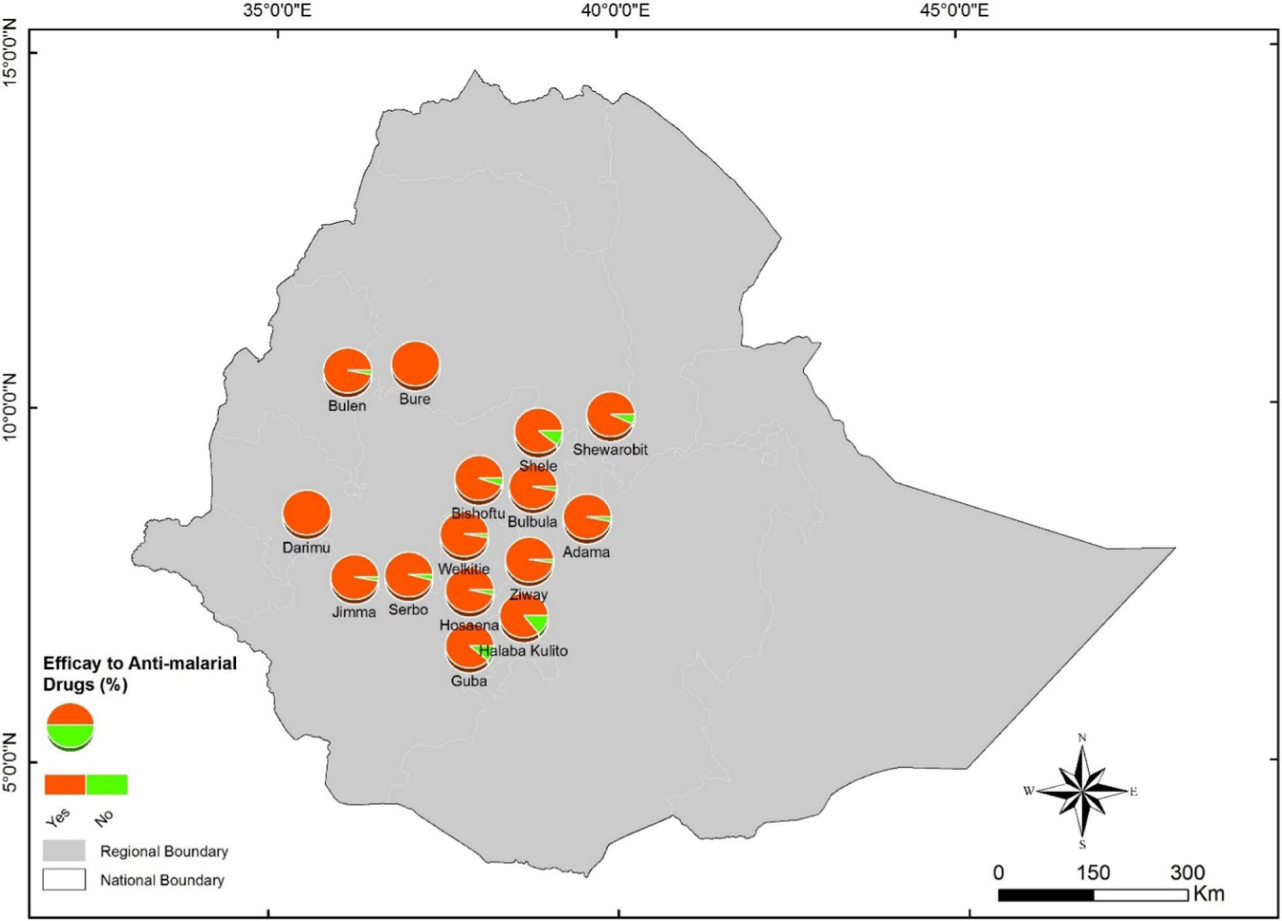
Map showing the distribution of study sites (n = 15) in Ethiopia where the efficacy of chloroquine against *P. vivax* malaria was investigated, 01 January 2000 to 31 March 2021

The 14 articles included in the current review reported data from 15 study sites and 29 treatment options. Five studies reported data from a single study site (Bishoftu/Debrezeit) in different years and seasons [20, 23, 26–28]. Two other studies reported data from another single study site (Adama/Naziret) [26, 27]. Figure 2 shows the distribution of the study sites from where the efficacy status of CQ has been reported (Fig. 2).

All 14 articles were written in English, and 10 of them reported results from single-arm, open-label, prospective cohort trials, each of which investigated the efficacy of CQ alone [22–24, 26, 27, 37–41]. One study was a randomized, double-blind, placebo-controlled trial [30], and the remaining three were randomized but open-label cohort trials [25, 28, 29]. These studies enrolled a total of 2144 patients (sample size of each individual study ranging from 27 to 145) of which 1495 were included for the efficacy evaluation of CQ alone. The remaining 649 patients were enrolled for investigation of the efficacy of combinations of different anti-malarial drugs, such as CQ plus PQ or AL plus PQ or AL alone. For 12 studies, the follow-up period was 28 days, while only two studies had a longer (42 days) follow-up period (Table 2). Upon enrolment, all patients were diagnosed by light microscopy, except in one study [30], where microscopy was supplemented with PCR. In four studies, genotyping of recurrent parasitaemia (LPTF) was further confirmed by PCR and blood drug level (CQ-DCQ (desethylchloroquine, a metabolite of CQ)) measurement [25, 28–30]. The remaining 10 studies either measured only blood drug levels [22, 37] or reported treatment failures without genotyping of recurrent parasitaemia, or they measured plasma levels on the day of parasite recurrence (as defined by microscopy only) [23, 24, 26, 27, 38, 40]. The majority of patients included in the individual studies were males (58.16%, n = 1247/2144) and aged > 14 years (80.48%, n = 1452/1804). Parasite and fever clearance were achieved before day 7 for most of the participants.

**Table 2:** Characteristics of studies included in in vivo anti-malarial drug efficacy studies against clinical vivax malaria in Ethiopia, 1 January, 2000 to 31 March, 2021

Study ID	Study period	Altitude (malaria transmission)	Study design	Key characteristics	Study district/ region	Treatment option and dose	Sample size (enrolled)	Gender (Male/ Female)	Median age in year (range/IQR)	Age range (year)			Follow-up days	Completed follow-up (N)	Treatment outcomes					
															TF			ACPR (n)	Ex	P. f./ mixed
										< 5	5–14	> 14			ETF		LTF			
Abreha et al [30]^a^	November- December, 2014	1800- 1900 (low)	A randomized double-blind placebo-controlled	Patients with normal glucose-6-phosphate dehydrogenase status with symptomatic *P*. *vivax* mono-infection	Bishoftu and Batu Health Centers, Oromia	CQ^b^ (25 mg/ Kg)	n = 104, Bishoftu (79), Batu (25),	61.5% (n = 64) males, 38.5% (n = 40) females	18 (IQR 10.5–19)	7	31	66	42	96	D28	1	3	92	2	2
														91	D42	1	17	77	7	2
						CQ (25 mg/ Kg) and PQ (0.25 mg/kg)	n = 100 Bishoftu (73), Batu (27),	70% (n = 70) males, 30% (n = 30) females	18 ( IQR 10–26)*	12	31	57	42	91	D28	0	0	91	3	3
														86	D42	0	0	85	12	4
						AL^c^	n = 102 Bishoftu (79), Batu (23)	66.7% (n = 68) males, 33.3% (n = 34) females	17 ( IQR 9–25)*	10	37	55	42	92	D28	0	11	81	0	0
														90	D42	0	27	62	11	0
						AL and PQ^d^ (0.25 mg/kg)	n = 92, Bishoftu (76), Batu (16)	55.4% (n = 50) males, 44.6% (n = 42) females	18 ( IQR 9–27)*	8	28	56	42	86	D28	0	2	84	0	0
														82	D42	0	5	77	10	0
Assefa et al. [26]	April to June, 2014	2177 (low)	A single arm open-label prospective cohort trial	Patients with *P. vivax* infection, who fulfilled the WHO inclusion criteria	Hossana Health Centre, Hadiya Zone, SNNPR	CQ (25 mg/ Kg)	n = 63	58.3% (n = 35) males, 41.7% (n = 25) females	23 median ( 4–59)	ND	ND	ND	28	60	0		2	58	3	1
Beyene et al. [24]	17 August to 19 December, 2014	1450 (moderate)	A single arm open-label, prospective cohort trial	Patients visiting a health centre and presented with clinically suspected malaria	Bullen Health Centre Metekel zone, Benshangul	CQ (25 mg/ Kg)	n = 76	68% (n = 47) males, 32% (n = 22) females	19 median (3–54)	20	15	34	28	69	0		2	67	3	2
Getachew et al [38]	May 2010 and December 2013	Moderate	A single arm open-label, prospective cohort trial	Patients attending outpatient clinics with signs and symptoms consistent with malaria, who fulfilled the WHO inclusion criteria	Shele in Arba Minch, Guba in Halaba, Batu in Adami Tulu and Shone in Eastern Badawacho Districts	CQ (25 mg/ Kg)	Shele (89)	65.2% (58) males, 34.8% (= 31) female)	10 (IQR, 4–18)	31	26	32	28	236	2		23	229	29	5
							Guba (52)	57.7% (n = 30) males, 42.3% (n = 22) females	12 (IQR, 4.5–22.5)	16	18	18								
							Batu (n = 57)	40.3% (n = 23) males, 59.3% (n = 34) females	12 (IQR, 6–20)	13	24	20								
							Shone (n = 90)	56.7% (n = 51) males, 43.3% (n = 39) females	6 (IQR, 4–9)	43	33	14								
Hwang et al. [25] ^1^	October, 2009-January, 2010	1900 (low)	A randomized but open label cohort study	Patients with *P. vivax* mono-infection, who fulfilled the WHO inclusion criteria	Bishoftu and Bulbula Health Centers/Oromia	CQ (25 mg/Kg)	n = 120	68.3% (n = 82) males, 31.7% (n = 38) females	18 median (1–65)	ND	ND	ND	42	108	D28	0	10	98	8	ND
														107	D42	NA	34	73	9	ND
						AL	n = 122	62.3% (n = 76) males, 37.7% (n = 46) females	11½ median (1–70)	ND	ND	ND	42	115	D28	0	28	86	12	ND
														113	D42	NA	47	66	13	ND
Kanche et al. [39]	10 February to 09 May, 2011	1780 (low)	A single arm open-label prospective cohort trial	Suspected malaria patients seeking medication who fulfilled the WHO inclusion criteria	Jimma town/Ormia	CQ (25 mg/ Kg)	n = 81	50.6% (n = 41) males, 49.4% (n = 40) females	6 months-60 years	7	25	49	28	74	1		1	74	7	ND
Ketema et al. [31]	October, 2007-January, 2008	1740 – 2660 (low)	A single arm open-label prospective cohort trial	Suspected malaria patients seeking medication, who fulfilled the WHO inclusion criteria	Serbo Health Center, Jimma zone, Oromia	CQ (25 mg/ Kg)	n = 84	60.7% (n = 51) males, 39.3%(n = 33) females	8 median (9/12–45)	ND	ND	21	28	78	0		3	78	2	3
Ketema et al. [23]	January to February, 2009	1726 (moderate)	A single arm open-label prospective cohort trial	Individuals seeking treatment for malaria at a Health Center during the study period and having *P. vivax* mono-infection	Halaba Kulito Health Center/ Halaba town/ SNPPR	CQ (25 mg/ Kg)	n = 87	42.6% (n = 36) males, 57.4% (n = 51) females	8 median (range 9/12–52)	35	13	39	28	80	4		7	69	7	1
Teka et al. [22]	June–August 2006	1900 (low)	A single arm open-label prospective cohort trial	Patients were recruited according to the WHO protocol for monitoring anti-malarial drug resistance	Bishoftu/Oromia	CQ (25 mg/ Kg)	n = 87	58.8% (n = 51) males, 41.4% (n = 36) females	16 median (8/12- 52)	18	ND	ND	28	83	0		4	79	1	3
Seifu et al. [40]	October 2013 to February 2014	1280 (moderate)	A single arm open-label prospective cohort trial	The study participants were individuals who had confirmed *P. vivax* mono-infection and who fulfilled the WHO inclusion criteria	Shawa Robit Health Centre,/ Amhara	CQ (25 mg/ Kg)	n = 87	71.3% (n = 62) males, 28.7% (n = 25) females	20 median (1–65)	29 (< 15 years)	ND	58	28	76	0		5	71	11	4
Shumbej et al. [27]	December, 2016—May, 2017	1710–1950 (low)	A single-arm open-label, prospective cohort trial	*P.vivax* mono-infected patients, fulfilled the inclusion criteria	Gurage zone/SNNPR	CQ (25 mg/ Kg)	n = 87	54.3%, (n = 45) males, 45.7% (n = 37) females	19 median (1.5–42)	10	16	55	28	81	0		2	81	5	1
Yeshanew et al. [41]	March and December, 2018	1700– 1900 (low)	A single-arm open-label, prospective cohort trial	Patients who were attending the outpatient clinics	Darimu District/ Oromia	CQ (25 mg/ Kg)	n = 128	64.5% (n = 42) males, 35.4% (n = 23) females	20 median (2–71)	3	24 (5–18 years)	38(> 18 years)	28	65	0		0	ND	ND	ND
		1300–1646 (moderate)			Bure District/ Oromia	CQ (25 mg/ Kg)		70% (n = 35) males, 30% (n = 15) females	23 median (5–60)	1	9 (5–18 years)	40 (> 18 years)	28	50	0		0	ND	ND	ND
Yeshiwondim et al. [28]^1^	January-August, 2003	1900 (low)	A randomized, open-label, cohort study	Patients with slide-confirmed malaria who presented to the outpatient settings	Debrezeit/Bishoftu and Nazareth/Adama towns/ Oromia	CQ (25 mg/ Kg)	n = 145	53.7% (n = 78) males, 46.2% (n = 67) females	20 median (4–65)	2	34	109	28	141	0		4	136	10	3
		1622 (moderate)				CQ (25 mg/ Kg) and PQ (0.25 mg/kg)	n = 145	54.5% (n = 79) males, 45.5% (n = 66) females	20 median (4–60)	1	34	110	28	141	1		0	141	11	3
Yohannes et al. [29]^a^	October 2004 to May 2005	1900 (low)	A randomized, open-label, cohort study	Patients fulfilled the inclusion criteria of WHO protocol for monitoring anti-malarial drug resistance	Bishoftu/Oromia	CQ (25 mg/ Kg)	n = 27	55.6% (n = 15) males, 44.4% (n = 12) females	21 median (IQR) (9.5–30)	0	ND	ND	28	21	0		3	18	6	ND
						AL	n = 36	50% (n = 18) males, 50% (n = 18) females	17 median (IQR) (10–25)	2	ND	ND	28	30	0		7	23	6	ND
		1622 (moderate)			Nazareth/Adama town	CQ (25 mg/ Kg)	n = 44	43.2% (n = 19) males, 56.8% (n = 25) females	17.5 median (IQR) (13–25)	5	ND	ND	28	36	0		2	34	8	ND
						AL	n = 52	40.4% (n = 21) males, 59.6% (n = 31) females	17 median (IQR) (7.6–23.3)	7	ND	ND	28	45	1		11	33	7	ND

ETF = early treatment failure, ACPR = adequate clinical and parasitological response, LTF = late treatment failure [this included late clinical treatment failure (LCTF) and late parasitological Treatment failure (LPTF)], WHO = World Health Organization, SNNPR = Southern Nations an d Nationalities People Region, Ex = Excluded from study (this includes those withdrawal, protocol violation, and loss to follow-up), ND = no data, NA = not applicable

^a^PCR-corrected

^b^CQ (25 mg/Kg) = (CQ treatment with a dose of 10, 10 and 5 mg/kg on days 0, 1 and 2, respectively)

^c^AL = (20 mg of artemether and 120 mg of lumefantrine)

^d^PQ = PQ (0.25 mg/kg daily dose over 14 days, from day 1–3 or from day 3–16 after treatment with CQ)

^e^Treatment efficiency for each anti-malarial drug was calculated by dividing ACPR (n) by those who completed (N) the follow-up (n/N) X 100

*IQR* = Interquartile range

All studies included in this meta-analysis reported the efficacy of anti-malarial drugs in clearing parasites and fever in *P. vivax-*infected patients. About 91.89 and 96.08% of the patients achieved parasite clearance on day 2 and day 3, respectively. Likewise, fever clearance was achieved for 80, 89.46 and 96.15% of the patients on day 1, day 2 and day 3, respectively. In all studies except one, complete parasite and fever clearance were achieved on day 7 [23], for each variable (Table 3). In the later [23] study, only 95.4% parasite clearance was recorded and no data are available for the status of fever clearance.

**Table 3:** Parasite and fever clearance reported from individual studies included in the anti-malarial drug efficacy study, 1 January, 2000 to 31 March, 2021

Study ID	Patients enrolled	Patients who completed follow-up	Patients with ACPR	Parasite clearance (%)^a^			Fever clearance (%)^a^			
				D2	D3	D7	D1	D2	D3	D7
Abreha et al. [30]										
CQ	104	96 (d28)/	92 (d28)/	95.2	98.1	100	89.5	100	100	100
		94 (d42)	77 (d42)							
CQ & PQ	100	94 (d28)/	94 (d28)/	95	100	100	100	100	100	100
		89 (d42)	89 (d42)							
AL	102	92 (d28)/	81 (d28)/	91.2	100	100	97.6	100	100	100
		90 (d42)	62 (d42)							
AL & PQ	92	90 (d28)/	84 (d28)/	100	100	100	97.4	97.4	97.4	100
		89 (d42)	77 (d42)							
Assefa et al. [26]	63	60	58	ND	ND	ND	ND	ND	ND	ND
Beyene et al. [24]	76	69	67	83	83	100	94.2	95.5	100	100
Getachew et al. [38]	288	236	229	93.8	100	100	ND	98.8	100	100
Hwang et al. [25]										
· CQ	120	108 (d28)/ 107 (d42)	98 (d28)/ 73 (d42)	94	98.1	100	44.1	77.8	90.4	100
AL	122	114 (d28)/ 113 (d42)	86 (d28), 66 (d42)	100	100	100	37.7	74.3	89.3	100
Kanche et al. [39]^b^	81	74	74	98.8	100	100	ND	93.8	100	100
Ketema et al. [31]^b^	84	78	78	88	88	100	65.4	70.5	89.7	91.7
Ketema et al. [23]^b^	87	80	69	95.4	95.4	95.4	ND	ND	ND	ND
Teka et al. [22]	87	83	79	ND	98	100	ND	ND	ND	ND
Seifu et al. [40]	87	76	71	91.3	100	100	ND	27.6	ND	ND
Shumbej et al. [27]	87	81	81	100	100	100	ND	100	100	100
Yeshanew et al. [41]	128	115	115	ND	75.6	100	ND	ND	71.7	100
Yeshiwondim et al. [28]										
CQ	145	141	141	80.1	97.9	100	59.6	97.2	100	100
CQ & PQ	145	136	136	72.6	99.3	100	94.9	98.5	100	100
Yohannes et al. [29]										
CQ	63	51	51	ND	ND	ND	90.1	100	100	100
AL	96	81	81	ND	ND	ND	89.8	100	100	100

*ND *No data available, *d28* day 28, *d42* day 42

^a^Parasite/fever clearance rates were taken from reports of individual studies

^b^When parasite or fever clearance only was reported, the rate was calculated by subtracting the percentage with parasites or fever from 100%

### Quality assessment of individual studies

The majority of studies, except for two [28, 29], fulfilled more than 50% or ≥ 4 quality domains out of the 7. All studies fulfilled at least two quality criteria: blinding of participants and personnel (performance bias) and blinding of outcome assessment (detection bias). In addition, all the studies met two quality criteria except for three studies that failed to fulfill attrition bias [25, 30, 38], and the other three studies that failed to fulfill reporting bias [22, 29, 41]. The most common quality criteria not fulfilled by the studies were the two selection biases: random sequence generation and allocation concealment. Only two studies [25, 30] fulfilled these two criteria (Additional file 1: Fig. S1a and b, and Additional file 3: Table S3).

### Main outcome of the meta-analysis

**Fig. 3:**
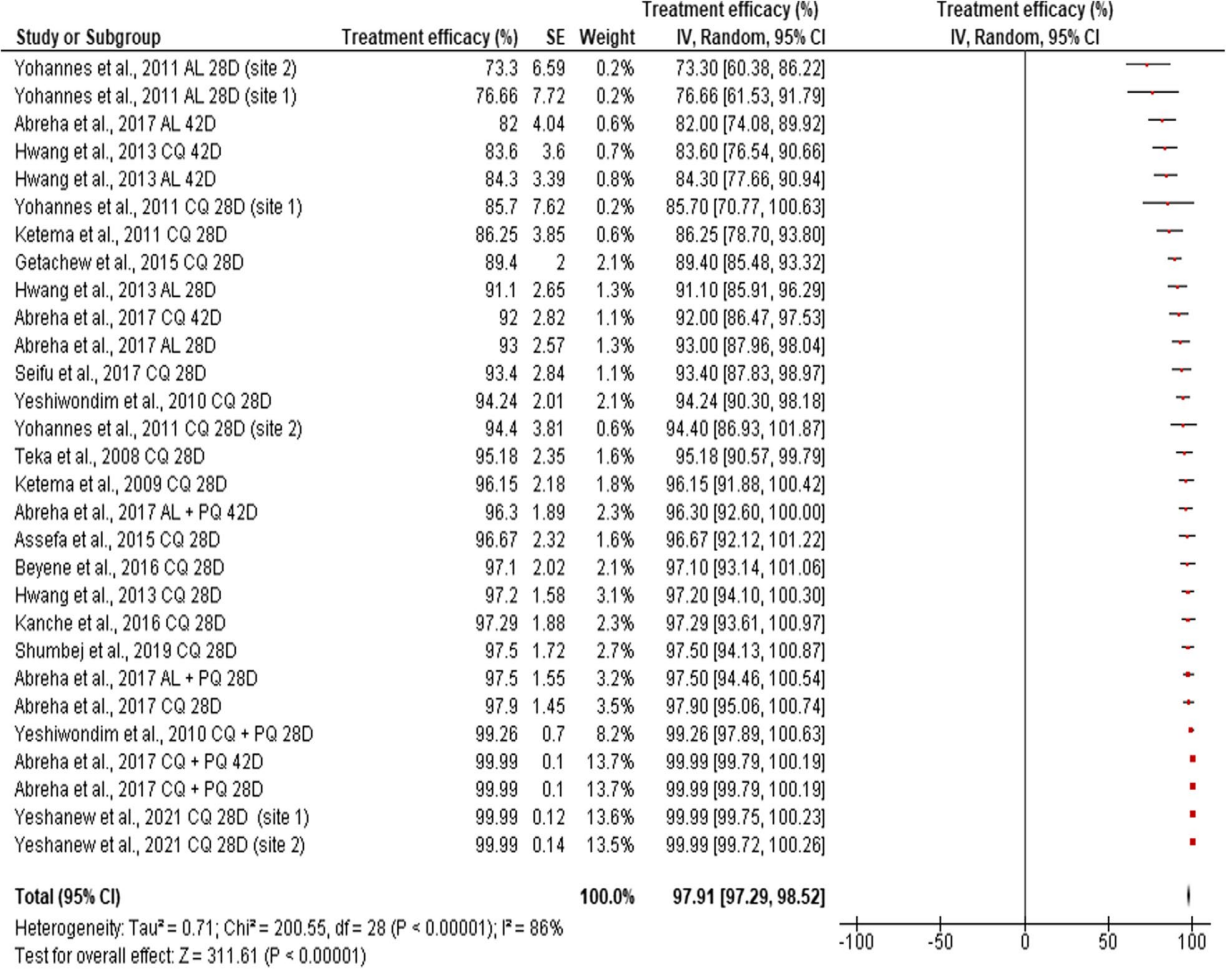
Individual and pooled estimates of the in vivo efficacy of antimalarial drugs against clinical *P. vivax* malaria infection in Ethiopia

The overall random, pooled, estimated efficacy of anti-malarial drugs against clinical vivax malaria in Ethiopia was 97.91% (95% CI: 97.29–98.52%), with a very significant high level of heterogeneity (*I*^2^ = 86%, p < 0.0001). Indeed, the efficacy of anti-malarial drugs against *P. vivax* across individual studies varied considerably, ranging from 73.3% for AL on day 28 [29] to 99.99% for CQ alone or CQ plus PQ on Day 28 [30, 41] (Fig. 3). Analysis of risk of publication bias among the studies included in the current review showed that there was publication bias as demonstrated by asymmetrical funnel plot, qualitatively, and significant bias quantitatively, as shown by Egger’s regression test (bias coefficient = − 12.86, *p* < 0.0001) (Additional file 2: Fig. S2).

**Fig. 4:**
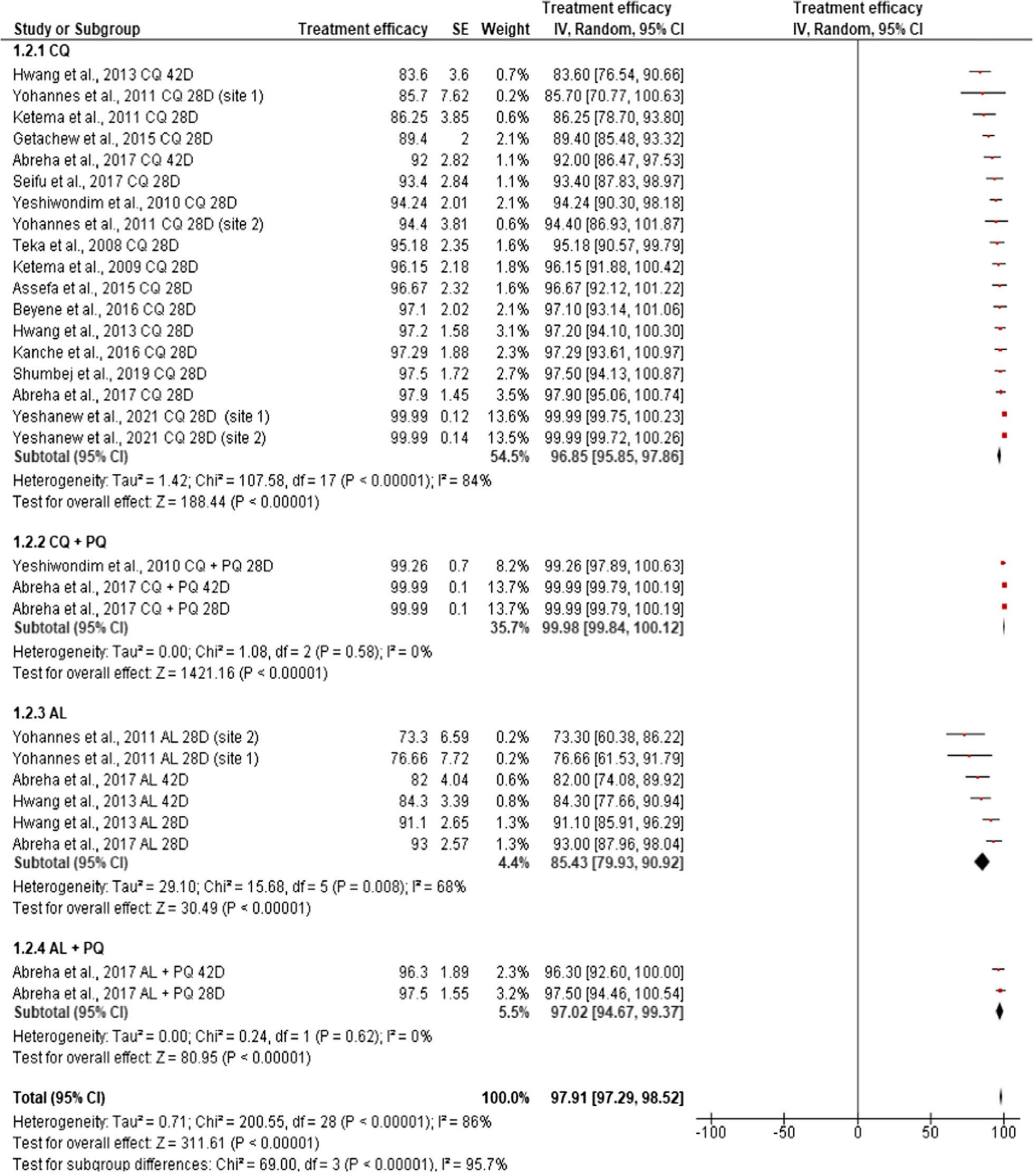
Pooled in vivo efficacy estimate of different anti-malarial drugs against clinical *P. vivax* malaria in Ethiopia

The pooled, estimated treatment efficacy of CQ alone, irrespective of the follow-up duration (28 or 42 days), was 96.85% (95% CI: 95.85–97.86, p < 0.0001), with a high level of heterogeneity (*I*^*2*^ = 84%). The combination of CQ plus PQ showed greater and consistent therapeutic efficacy (99.98%, 95% CI: 99.84–100.12, *I*^*2*^ = 0%) than CQ alone. On the other hand, AL alone, irrespective of post-treatment follow-up periods, showed significantly the lowest (85.43%, 95% CI: 79.93–90.92, p = 0.008) efficacy against *P. vivax* compared to other treatment options, but its supplementation with PQ resulted in enhanced efficacy (97.02%, 95% CI: 94.67–99.37, p = 0.62). The efficacy of the different anti-malarial drugs against clinical vivax malaria considered in the current meta-analysis did appear to significantly affect the pooled estimate prevalence of *P. vivax* (*χ*^2^ = 69, df = 3, p < 0.0.001, *I*^2^ = 95.7%) (Fig. 4).

**Fig. 5:**
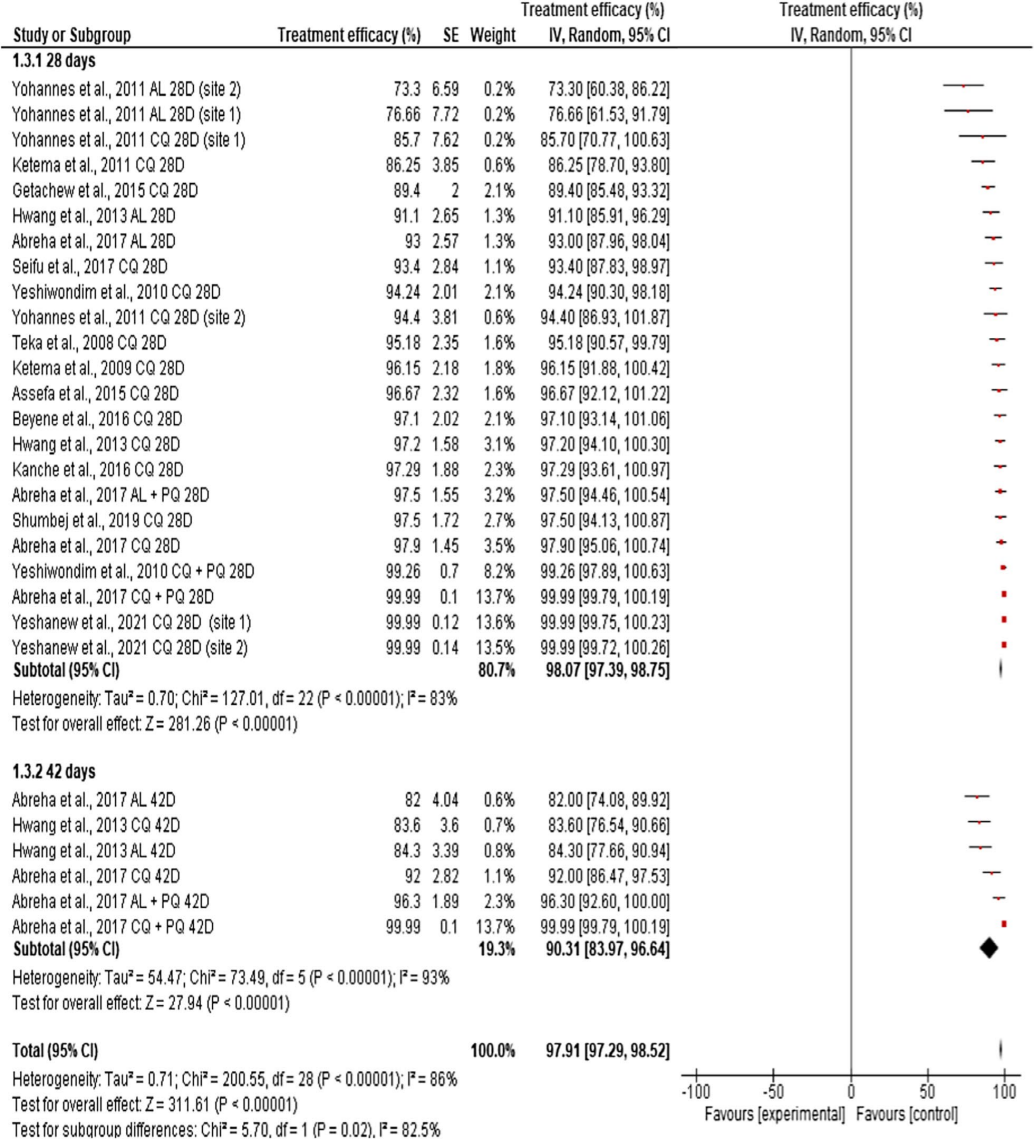
Pooled in vivo efficacy estimate of different antimalarial drugs against clinical *P. vivax* malaria in Ethiopia with respect to post-treatment follow-up periods

Differences in the duration of follow-up (28 days *vs* 42 days) significantly affected the overall pooled efficacy of anti-malarial drugs against *P. vivax* (*χ*^2^ = 5.70, df = 28, p = 0.02, and *I*^*2*^ = 82.5%). Treatment efficacy of anti-malarial drugs reported on day 28 showed significantly higher efficacy (98.07%, 95% CI: 97.39–98.52%, p < 0.001) compared to the efficacy reported on day 42 (90.31%, 95% CI: 83.97–96.64%, p < 0.0001) (Fig. 5).

### Transmission intensity

**Fig. 6:**
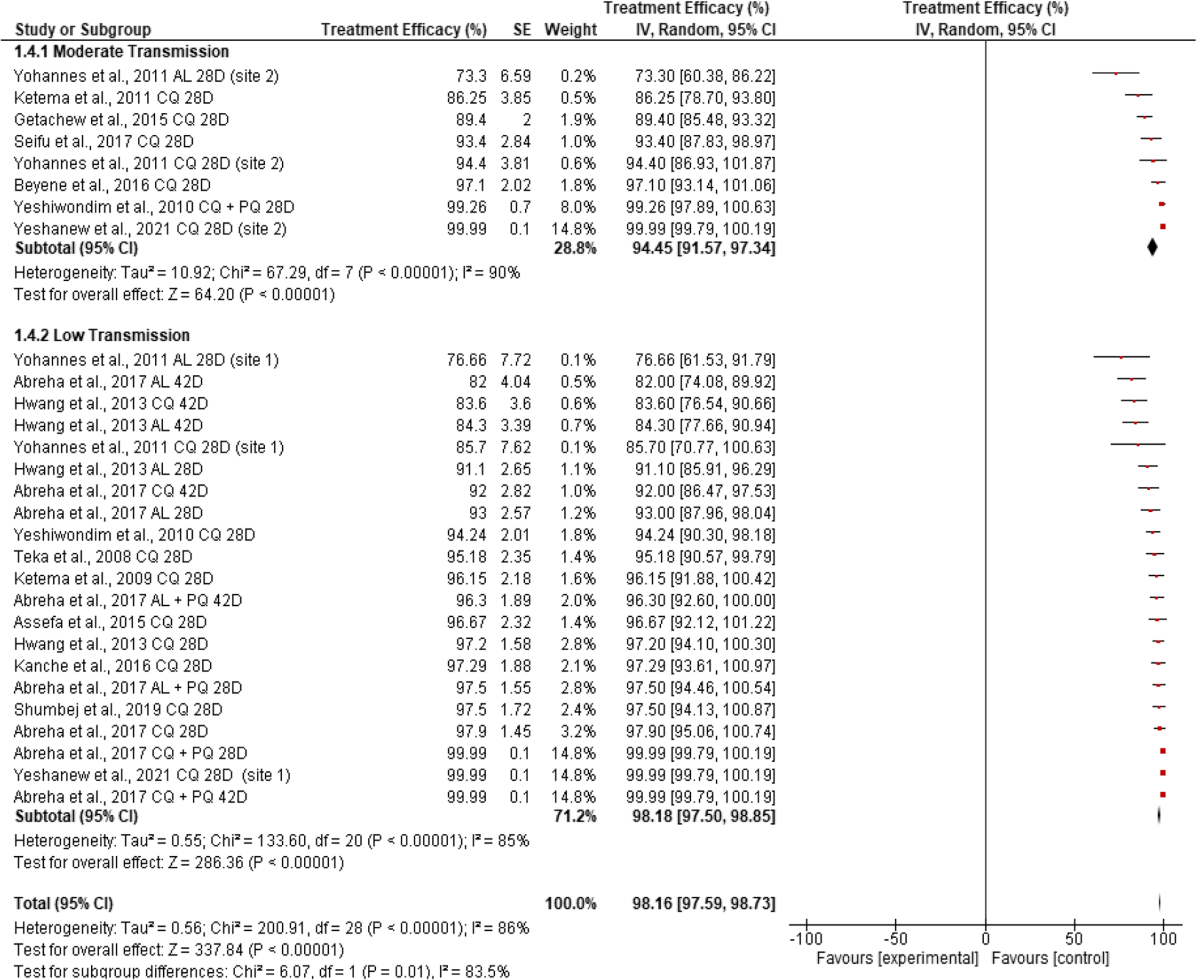
Pooled in vivo efficacy of anti-malarial drugs against clinical *P. vivax* malaria infection in Ethiopia at different malaria transmission settings

Pooled efficacy in altitudinally intermediate transmission settings was significantly lower (94.45%, 95% CI: 91.57–97.34, p < 0.001) than in altitudinally lower transmission areas (98.18%, 95% CI: 97.5–98.85). The transmission setting significantly affected the overall calculated efficacy of anti-malarial drugs (*χ*^2^ = 6.07, df = 1, *I*^*2*=^ 83.5%, p = 0.01). None of the included studies reported data from high malaria transmission settings in Ethiopia (< 1000 m altitude) (Fig. 6).

**Fig. 7:**
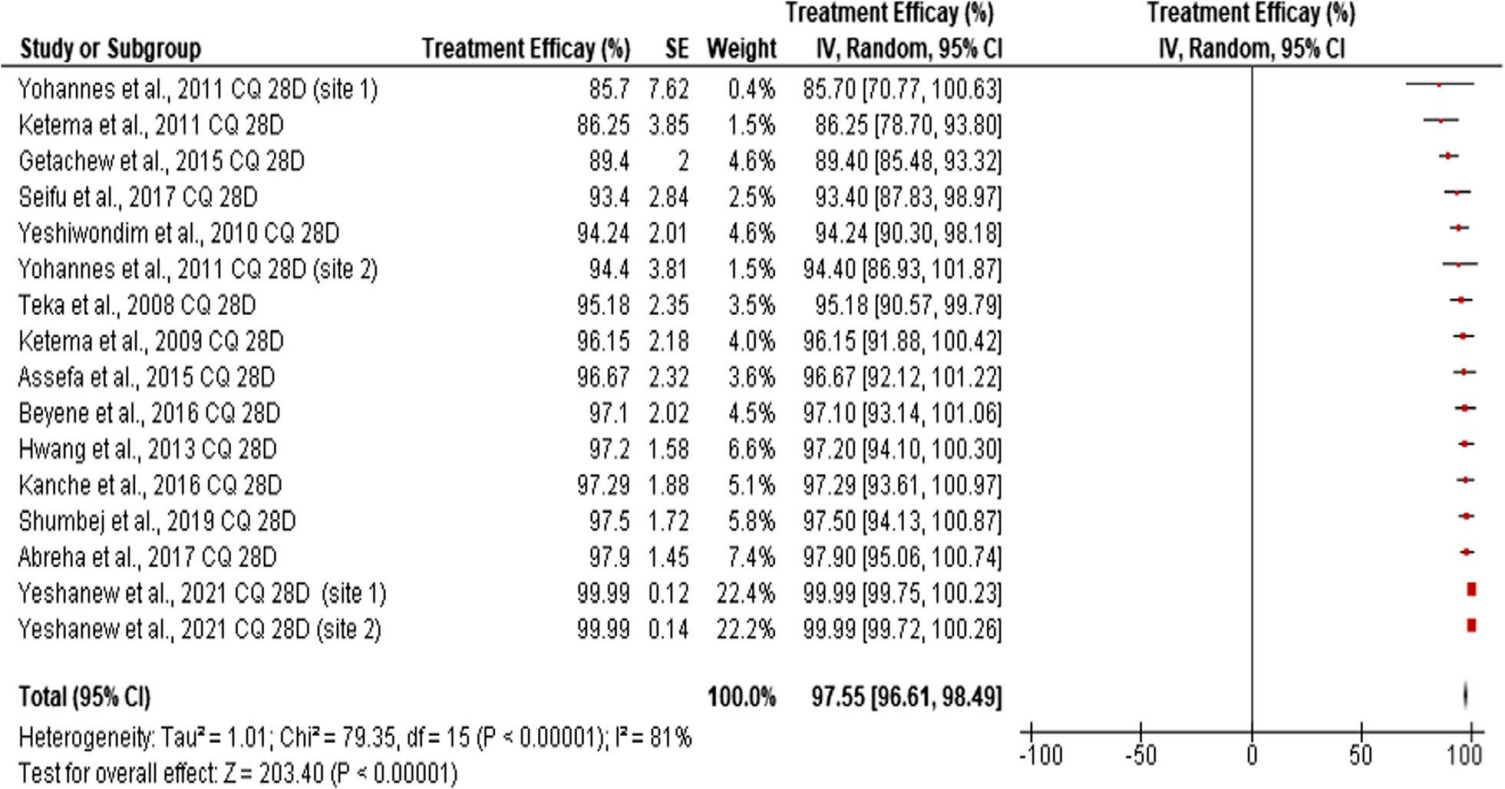
Pooled estimated efficacy of CQ against clinical *P. vivax* malaria in Ethiopia on day 28

Similarly, the estimated efficacy reported for CQ alone on day 28 showed slight improvement (97.55, 95% CI: 96.61–98.49) as compared to the overall pooled estimated efficacy reported for all treatment options (28 and 42 days) (96.85%, 95% CI: 95.85–97.86) (Fig. 7).

**Fig. 8:**
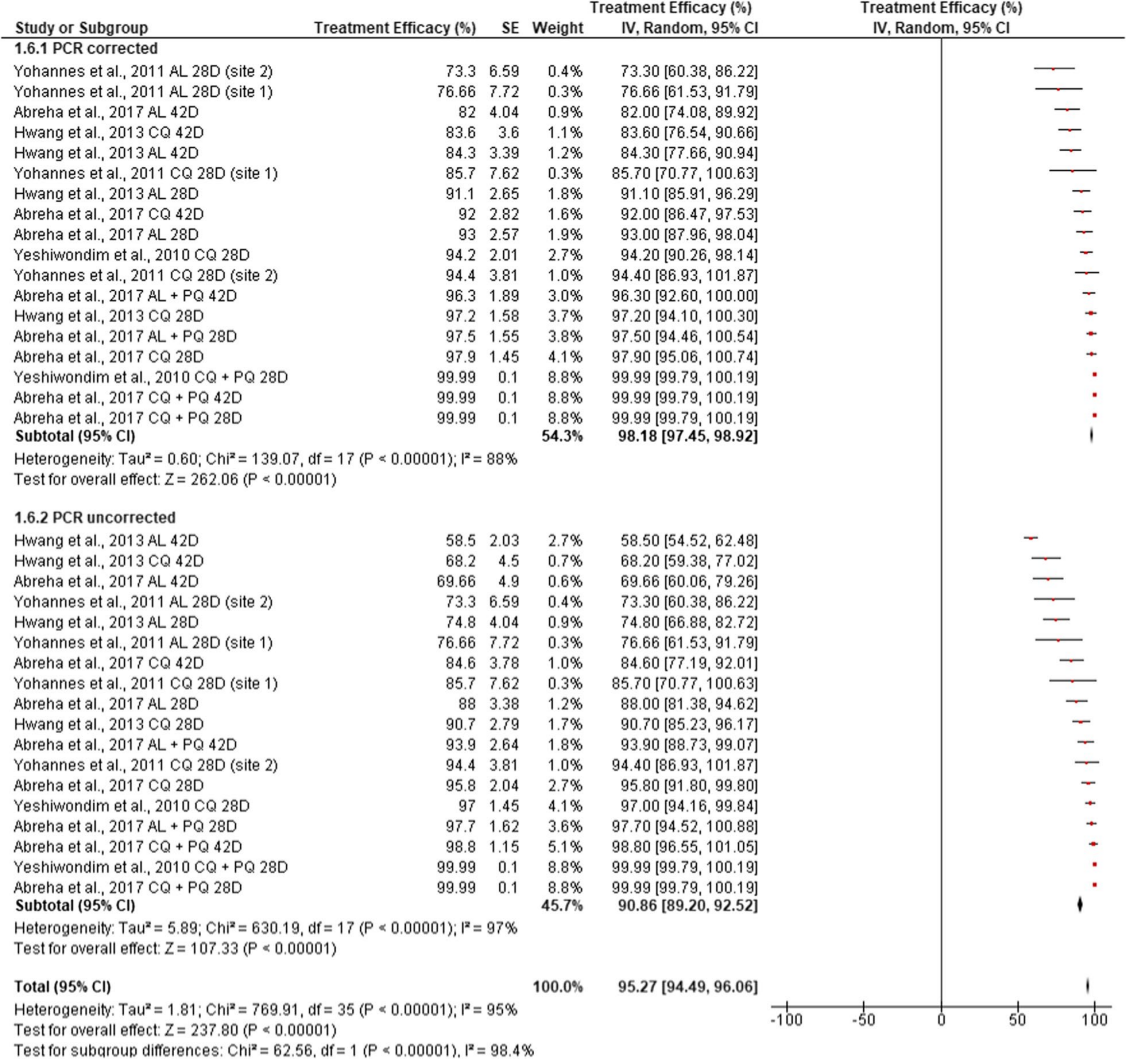
Pooled in vivo efficacy estimates for different antimalarial drugs for treating clinical *P. vivax* malaria infection in Ethiopia, with respect to recurrent parasitemia confirmatory testing

The presence or absence of results of confirmatory molecular tests for recurrent parasitaemia (only those studies with matching or paired information for PCR-corrected and PCR-uncorrected results) revealed significant heterogeneity and differences between the pooled efficacy of anti-malarial drugs (*χ*^2^ = 62.56, df = 1, *I*^*2*^ = 98.4%, p < 0.0001). There was significantly reduced therapeutic efficacy as regards PCR-uncorrected efficacy reports (90.86, CI: 89.20–92.52, P < 0.0001) as compared to treatment failures that were PCR-corrected (98.18 (95%, CI: 97.45–98.92) (Fig. 8).

## Discussion

This systematic review and meta-analysis was conducted with the aim of reviewing studies that reported results of in vivo anti-malarial drug therapy for clinical vivax malaria in Ethiopia. Those studies that focused on the therapeutic efficacy of different anti-malarial drugs against *P. vivax,* and fulfilled the WHO-recommended efficacy testing procedures, and which were published between 1 January, 2000 and 31 March, 2021 were incorporated in this analysis. While all the included research had investigated the susceptibility of *P. vivax* to CQ, four of the studies additionally evaluated the potential efficacy of other anti-malarial drugs or drug combinations against this parasite species. These treatment options included AL, AL plus PQ separately, and CQ plus PQ. Findings from the meta-analysis showed that, the overall aggregated anti-*P. vivax* treatment efficacy estimated for these anti-malarial drugs was excellent, at 97.91% (95% CI: 97.29–98.52%), which is well above the recommended WHO threshold for anti-malarial efficacy (≥ 90%). This pooled, estimated efficacy was affected by the treatment options, duration of the follow-up, transmission intensity, and confirmatory tests for the recurrent parasitaemia. In all the analyses, there was substantial unexplained, high heterogeneity within the studies included. Hence, the validity of the effect estimated for each sub-group is uncertain as individual studies varied in terms of treatment type, follow-up duration, and confirmatory tests for the efficacy.

The main drug investigated in all individual studies included in this review was CQ, the current first-line treatment for vivax malaria in Ethiopia. For this drug, the pooled, estimated efficacy was 96.85% (95% CI: 95.85–97.86). The slight efficacy improvement observed on day 28 post-treatment for CQ (97.55%, 95%CI: 96.61–98.49) in comparison with the greater overall, pooled, estimated efficacy for CQ (on day 28 and day 42) could be attributed to the drug’s longer, indirectly monitored elimination time, which could reflect continued protection against re-infection and suppression of early relapses [42]. But, as the duration of follow-up increases, it is expected that the blood drug level will drop to below the MEC. At this level, it can no longer guarantee protection from relapses or re-infections. Re-activation of hypnozoites in the liver, leading to relapse, is one of the typical features of vivax malaria. The re-activation of these hypnozoites may occur within as short a period as two weeks or after as long as 10 months following the primary infection [43]. Although the exact re-activating factors are not understood, environmental conditions and host biology have been hypothesized as contributing factors [43, 44]. Hence, the risk and frequency of relapse are much higher in tropical regions than in temperate regions [43]. The reduced efficacy observed on day 42 (90.31%) of the follow-up period compared to day 28 (98.07%) might be attributable to a relapse of the previous clinical episodes or it might be the result of re-activation of pre-existing hypnozoites that were present. Also, re-infection with a new parasite inoculum is a possibility because transmission frequency by mosquitoes can be high in intense malaria transmission settings. This was further revealed by one of the studies where CQ efficacy dropped to 83.6% on day 42 from an efficacy of 97.9% on day 28 [28]. However, the fact that four studies reported PCR-corrected treatment failure [25, 28, 29], and the finding of blood drug levels (CQ-DCQ) above the MEC (100 ηg/ml) in six of the studies on the day of recurrence [22, 25, 28–30, 37] implies the possibility of emergence and expansion of CR*Pv* in the country.

Most of the articles included in this review reported data from studies conducted in north-central and central Rift Valley areas, and southwest of Ethiopia. Nine out of 16 studies reported data from north-central and central Rift Valley regions (Bishoftu, Bulbula, Adama, Halaba Kulito, Guba, Shele, Shewa Robit, Batu/Ziway), where *P. vivax* is the dominant malaria parasite and accounts for ~ 50–70% [22, 45–47] of infections. According to the recent malaria stratification and mapping of the country [35], these areas are considered as moderate (altitude range from 1000 to 1750 m) *P. vivax* transmission areas. The rest of the studies were conducted in the southwest of the country (Serbo, Jimma, Darimu, Bure, Hossana, Gurage zone, Bullen), where *P. falciparum* is the dominant malaria parasite, and *P. vivax* accounts for only < 40% of total infections (‘low *P. vivax* transmission areas’) [24, 26, 27, 37]. Studies have shown that in areas where the vivax malaria burden/transmission is higher, the parasite can easily develop resistance (or exhibit decreased sensitivity) to CQ [48, 49]. In agreement with this fact, the estimated efficacy of CQ in areas where vivax malaria prevalence accounts for about ~ 50–70% of all malaria infections was 94.45% (95% CI: 91.57–97.34) [22, 23, 25, 28–30]. However, in areas where *P. vivax* has been less prevalent (accounting for < 40% of the total number of infections), anti-malarial drugs have shown excellent efficacy, estimated at 98.18%, (95% CI: 97.5–98.85) [24, 26, 27, 31, 39, 41]. Among the 14 studies included in the current review, 11 (n = 11) of them reported PCR-uncorrected treatment efficacy. These studies have shown lower estimated efficacy compared to the PCR-corrected ones. An important determinant of day 28 PCR-uncorrected efficacy is the intensity of malaria transmission in the study area. In high/moderate transmission settings, some of the drugs, such as AL, will cease protecting after 15–20 days as the half-life of lumefrantrine is short compared to that of CQ [50], and increases risk of re-infection or activation of hepatic hypnozoites.

CQ has been in use for more than 60 years for the treatment of vivax malaria in Ethiopia [10]. Although the first evidence of decreasing efficacy against the parasite was documented more than two decades ago [9], it remains the first-line drug for treatment of uncomplicated *P. vivax* infection. Findings in this review further highlight the emergence and wider spread of CQ-resistant *P. vivax* strains in different parts of the country [25–27]. Many studies investigated markers for CQ resistance in *P. vivax*, mainly mutation of the genes responsible for the observed resistance, including *Pvmdr*-1 and *Pvcrt*-0, from the same study sites (Jimma, Halaba, Omo Nada, Arbaminch, Hawasa) where the development and expansion of CR*Pv* in the country was confirmed [51–55]. In these studies of mutations in the *Pvcrt* and *Pvmdr*-1genes, two of the non-synonymous mutations at Y976F and F1076L were identified in the majority of the CQ-resistant *P. vivax* isolates.

Treatment with CQ and PQ, which offers a blood schizontocidal and hypnozoitocidal therapy (CQ 25 mg/kg for 3 days plus PQ 0.25 mg/kg for 14 days) significantly improved the therapeutic efficacy to 99.99%, even under conditions of longer follow-up, although the observation was based on few studies. As has been indicated above, the efficacy of the blood schizontocidal drug CQ can slowly diminish and fall to below MEC with loss of protection against re-infection with new parasites or relapse of the initial infection. Its supplementation with PQ could help to clear hypnozoites from the liver and protect against relapses. Besides its efficient hypnozoitocidal activity, reports showed that PQ could enhance the efficacy of CQ even in a setting where CR*Pv* has become a serious concern [56–59]. The major risk of using PQ is the possibility of severe haemolysis in individuals with G6PD deficiency [16, 17]. Hence, the requirement for prior checking of the status of this enzyme in a patient would make the feasibility of its easy use very challenging. At the time of this review, PQ is not part of the national anti-malarial treatment policy for routine use in respect of vivax malaria patients in Ethiopia.

AL, on the other hand, which is first-line treatment for falciparum malaria in Ethiopia [10], showed significantly lower efficacy against *P. vivax* (85.43%) irrespective of the duration of follow-up. Because of its shortest elimination half-life (3–6 days), and its fastest-dropping concentration to below MEC, this drug combination (AL) could not protect patients from any relapse or re-infection that might appear as of the 21st day after initial infection in tropical regions [60]. Despite its use for longer periods and evidence for the emergence and expansion of CR*Pv* in different regions of Ethiopia, CQ has still shown superior efficacy over AL for the treatment of vivax malaria in Ethiopia.

Recurrent *P. vivax* parasitaemia following treatment is an indicator of treatment failure. However, classifying this treatment failure into recrudescent or new infections that appeared during follow-up in high malaria transmission areas is crucial, albeit currently challenging [61]. PCR-correction or adjustment is required to prevent misinterpretation, mainly overestimation of the efficacy of drugs. PCR-uncorrected efficacy reports of recurrent parasitaemia after treatment as re-infection might be mistakenly considered as recrudescence when it is not the case [61]. This could lead to reporting of low cure rates and falsely make efficacious drugs look less effective. In the current review, four studies [25, 28–30] comprising 18 different treatment options with paired PCR-corrected and PCR-uncorrected results were separately analysed. In agreement with the above premises, the finding showed that the reported PCR-corrected efficacies were significantly higher (98.18 (95%, CI: 97.45–98.92)) than the PCR-uncorrected efficacies (90.86, CI: 89.20–92.52, p < 0.0001), which indicates the importance of using confirmatory molecular tests for any in vivo anti-malarial drug efficacy evaluation and reporting of vivax malaria.

## Limitations of the study

Some of the limitations of this analysis were: firstly, the number of studies that focused on in vivo anti-malarial drug efficacy testing against *P. vivax* in Ethiopia and which were finally selected for inclusion were few. Secondly, the studies incorporated in the review lacked consistency in respect of follow-up: in some of the studies, the primary endpoint was 28 days, whereas it was 42 days for others. Such discrepancies had a significant effect on the pooled estimate of efficacy of anti-malarial drugs for vivax malaria. Variation in the experimental design among the studies also created significant challenges as regards using similar tools for efficacy analysis and drawing clear conclusions concerning the efficacy estimates for the drugs. In most of the studies considered for this review, recurrent parasitaemias were neither genotyped nor compared with the pre-treatment parasitaemia, and recurrent parasites were not checked to evaluate whether they were perhaps due to re-infection with different strains of the parasite or possibly due to the result of relapse involving different genotype. In addition, most of the studies were focused on CQ efficacy testing. For other anti-malarial drugs or combinations, the available studies were insufficient to make comparisons, and to assess their effects on the overall, estimated pooled efficacy. Furthermore, some published studies included only short methodological and results sections, and it was difficult to extract relevant information/data for further analysis. High heterogeneity of study design, which requires further explanation and determination of the causes was another challenge encountered during the course of the current review processes.

## Conclusion

The efficacy of different anti-malarial drugs evaluated for the treatment of vivax malaria in Ethiopia has shown a wide range of variability. Drug efficacy was mainly affected by the treatment options, duration of follow-up, malaria transmission settings, and the recurrent parasitaemia confirmation procedures. Those anti-malarial drugs supplemented with PQ showed excellent efficacy (up to 99.9%) when compared to any other options irrespective of the duration of follow-up and treatment options. By contrast, AL alone showed significantly lower efficacy against clinical vivax malaria. Regardless of strong evidence for the decreasing efficacy of CQ, the first-line regimen for the treatment of vivax malaria in Ethiopia, this review shows that CQ still has good efficacy in the country, and that urgent replacement with other anti-malarial drugs may not be needed nor justifiable, at least in the short term. On the other hand, supplementation of CQ with PQ could enhance efficacy, and might serve as an optional regimen for the treatment of vivax malaria in the country, provided a patient’s safety in terms of haemolysis risk is minimized. Regular monitoring and continuous surveillance of the efficacy of CQ remains necessary to minimize the risk of the spread of CQ-resistance.

## Supplementary Information

**Additional file 1: Fig. S1**. Risk of bias assessment graph (a) and summary (b) of studies on in vivo efficacy of antimalarial drugs against P. vivax malaria in Ethiopia

**Additional file 2: Fig. S2**. Funnel plot for publication bias assessment of studies on in vivo efficacy of antimalarial drugs against clinical P. vivax malaria in Ethiopia.

**Additional file 3: Table S1**. Summary of search keywords/terms.** Table S2**. Excluded studies and reasons for exclusion of studies on in vivo efficacy of anti-malarial drugs against clinical vivax malaria in Ethiopia.** Table S3**. ROB-2 tools for randomized and non-randomized studies on in vivo efficacy of anti-malarial drugs against clinical vivax malaria in Ethiopia

## Data Availability

All data supporting the conclusions are included in the manuscript.

## Abbreviations

ACPR: Adequate clinical and parasitological response
AL: Artemether lumefantrine
CQ: Chloroquine
CR*P*v: Chloroquine-resistant *Plasmodium vivax*
CT: Clinical trials
CQ-DCQ: Chloroquine-desethylchloroquine
ETF: Early treatment failure
FMoH: Federal Ministry of Health of Ethiopia
G6PD: Glucose-6-phosphate dehydrogenase
Hb: Haemoglobin
IQR: Interquartile range
IRS: Indoor residual spraying
ITN: Insecticide treated bed nets
LTF: Late treatment failure
LCTF: Late clinical treatment failure
LPTF: Late parasitological treatment failure
MEC: Minimum effective concentration
PQ: Primaquine
PRISMA: Preferred Reposting Items for Systematic Reviews and Meta-analyses
RCT: Randomized controlled trials
SE: Standard error
TF: Treatment failure
WHO: World Health Organization
