# Supplementary figures and images for: In vivo efficacy of anti-malarial drugs against clinical Plasmodium vivax malaria in Ethiopia: a systematic review and meta-analysis
Source: Malar J. 2021 Dec 24;20:483. doi: 10.1186/s12936-021-04016-2 (PMC8709955; doi:10.1186/s12936-021-04016-2)

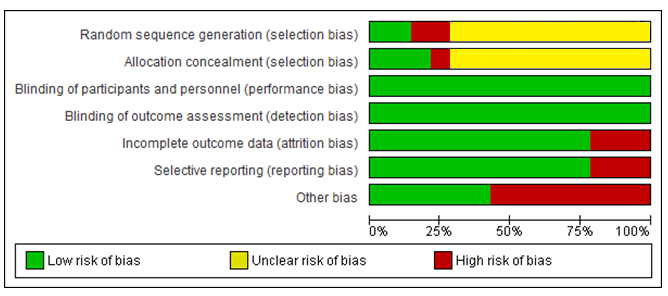


(a)


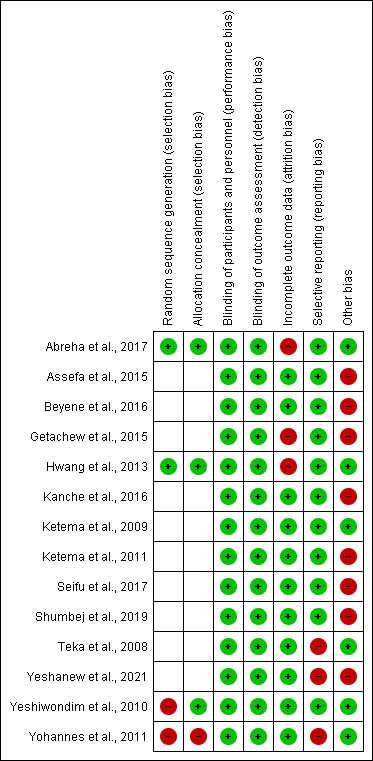


(b)

Supplement: Supplementary file 1 — Additional file 1: Fig. S1. Risk of bias assessment graph (a) and summary (b) of studies on in vivo efficacy of antimalarial drugs against P. vivax malaria in Ethiopia [file 12936_2021_4016_MOESM1_ESM.docx]

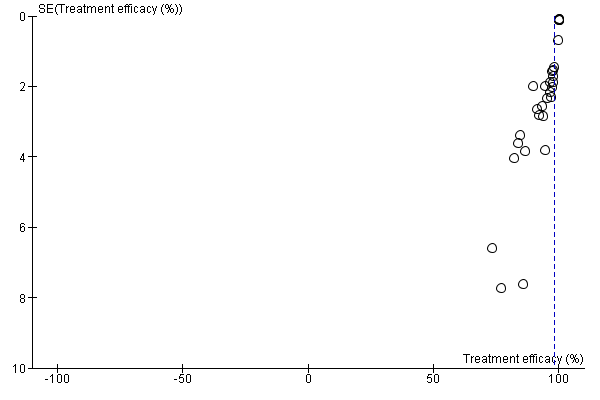

Supplement: Supplementary file 2 — Additional file 2: Fig. S2. Funnel plot for publication bias assessment of studies on in vivo efficacy of antimalarial drugs against clinical P. vivax malaria in Ethiopia. [file 12936_2021_4016_MOESM2_ESM.docx]
