# Supplementary material for: In vivo efficacy of anti-malarial drugs against clinical Plasmodium vivax malaria in Ethiopia: a systematic review and meta-analysis
Source: Malar J. 2021 Dec 24;20:483. doi: 10.1186/s12936-021-04016-2 (PMC8709955; doi:10.1186/s12936-021-04016-2)
Supplement: Supplementary file 3 — Additional file 3: Table S1. Summary of search keywords/terms. Table S2. Excluded studies and reasons for exclusion of studies on in vivo efficacy of anti-malarial drugs against clinical vivax malaria in Ethiopia. Table S3. ROB-2 tools for randomized and non-randomized studies on in vivo efficacy of anti-malarial drugs against clinical vivax malaria in Ethiopia [file 12936_2021_4016_MOESM3_ESM.docx]

**Supplementary Table 1** **Summary of search keywords/terms**

| **Database** | **Search strategy** |
| --- | --- |
| PubMed= 1057 | ("chloroquine"[MeSH Terms] OR "chloroquine"[All Fields]) OR ("primaquine"[MeSH Terms] OR "primaquine"[All Fields]) OR (("artemether, lumefantrine drug combination"[MeSH Terms] OR ("artemether"[All Fields] AND "lumefantrine"[All Fields] AND "drug"[All Fields] AND "combination"[All Fields]) OR "lumefantrine drug combination artemether"[All Fields] OR ("artemether"[All Fields] AND "lumefantrine"[All Fields]) OR "artemether and lumefantrine"[All Fields]) AND ("plasmodium vivax"[MeSH Terms] OR ("plasmodium"[All Fields] AND "vivax"[All Fields]) OR "plasmodium vivax"[All Fields]) AND ("ethiopia"[MeSH Terms] OR "ethiopia"[All Fields])) AND ("2000/01/01"[PubDate] : "2021/03/31"[PubDate]) |
| Web of Science (Core Collection) = 87  Citation Indexes: SCI-EXPANDED, SSCI, A&HCI, CPCI-S, CPCI-SSH, BKCI-S, BKCI-SSH, ESCI, CCR-EXPANDED, IC. | TS=(antimalarial OR Plasmodium OR malaria*) AND (TS=Ethiopia* OR CU=Ethiopia) AND PY=2000-2020 |
| Scopus = 132 | TITLE-ABS-KEY (Anti-malarial OR Plasmodium OR malaria*) AND (TITLE-ABS-KEY(Ethiopia*) OR AFFILCOUNTRY (Ethiopia)) AND PUBYEAR AFT 1999 AND PUBYEAR BEF 2021 |
| Clinical Trial.gov = 18 | Antimalarial drugs in Title Abstract Keyword AND Plasmodium vivax or vivax or plasmodium vivax or malaria in Title Abstract Keyword AND "Ethiopia" in Title Abstract Keyword |
| African Journals Online (AJOL) = 2 | Manually searched |

**Supplementary Table 2 Excluded studies and reasons for exclusion of studies on *in vivo* efficacy of anti-malarial drugs against clinical vivax malaria in Ethiopia**

| S/N | Authors | Title | Year | Journal | Reason for exclusion |
| --- | --- | --- | --- | --- | --- |
| 1 | Auburn et al | Genomic Analysis of *Plasmodium vivax* in Southern Ethiopia Reveals Selective Pressures in Multiple Parasite Mechanisms | 2019 | *Journal of Infectious Disease* | There is no intervention and outcomes measurement. Only genes responsible for drug resistance were investigated |
| 2 | Degefa, T. | In vivo sulphadoxine-pyrimethamine sentitivity study Tigray region, southern zone, Alamata town, September-November 2001 | 2004 | *Ethiopian Medical Journal* | Other *Plasmodium* spp, |
| 3 | Ebstie et al | Tafenoquine and its potential in the treatment and relapse prevention of *Plasmodium vivax* malaria: the evidence to date | 2016 | [*Drug Design, Development and Therapy*](https://www.dovepress.com/drug-design-development-and-therapy-journal) | There is no intervention and outcomes measurement. |
| 5 | Golassa et al. | Polymorphisms in chloroquine resistance-associated genes in *Plasmodium vivax* in Ethiopia | 2015 | *Malaria Journal* | There is no intervention and outcomes measurement. Only genes responsible for drug resistance were investigated |
| 6 | Gurmu et al., 2018 | Treatments used for malaria in young Ethiopian children: a retrospective study | 2018 | *Malaria Journal* | There is no intervention and outcomes measurement. |
| 7 | Hailemeskel et al. | Prevalence of *Plasmodium falciparum* *Pfcrt* and *Pfmdr1* alleles in settings with different levels of *Plasmodium vivax* co-endemicity in Ethiopia | 2019 | *International Journal for Parasitology: Drugs and Drug Resistance* | There is no intervention and outcomes measurement. |
| 8 | Heuchert et al. | Molecular markers of anti-malarial drug resistance in southwest Ethiopia over time: Regional surveillance from 2006 to 2013 | 2015 | *Malaria Journal* | There is no intervention and outcomes measurement. Only genes responsible for drug resistance were investigated |
| 9 | Kefyalew et al., | Efficacy of six-dose regimen of artemether-lumefantrine for the treatment of uncomplicated falciparum malaria, three years after its introduction into Ethiopia | 2009 | *Parasite* | Other plasmodium species, *P.falciparum* |
| 10 | Lacerda et al. | Single-dose tafenoquine to prevent relapse of *Plasmodium vivax* malaria | 2019 | *New England Journal of Medicine* | For clinical trial purpose, not recommended drug in the country |
| 4 | Lemma et al., | Cost-effectiveness of three malaria treatment strategies in rural Tigray, Ethiopia where both *Plasmodium falciparum* and *Plasmodium vivax* co-dominate. | 2011 | *Cost Eff Resour Alloc*. | The study didn’t follow the WHO procedure for drug efficacy testing |
| 11 | Lo et al., | Transmission dynamics of co-endemic *Plasmodium vivax* and *P. falciparum* in Ethiopia and prevalence of antimalarial resistant genotypes | 2017 | PLoS Neglected Tropical Disease | There is no intervention and outcomes measurement. Only genes responsible for drug resistance were investigated |
| 12 | Mavrogordato et al., | A cluster of *Plasmodium vivax* malaria in an expedition group to Ethiopia: Prophylactic efficacy of atovaquone/proguanil on liver stages of *P. vivax* | 2012 | *Journal of Infection* | There is no intervention, and its outcomes were not measurement |
| 13 | Mekonnen et al. | Return of chloroquine-sensitive *Plasmodium falciparum* parasites and emergence of chloroquine-resistant *Plasmodium vivax* in Ethiopia | 2014 | *Malaria Journal* | There is no intervention and outcomes measurement. |
| 14 | [Meltzer et al., 2018](javascript:;) | Vivax Malaria Chemoprophylaxis: The Role of Atovaquone-Proguanil Compared to Other Options. | 2018 | *Clinical Infectious Diseases,* | There is no intervention, and its outcomes were not measurement |
| 15 | Mula et al. | Detection of high levels of mutations involved in anti-malarial drug resistance in Plasmodium falciparum and *Plasmodium vivax* at a rural hospital in southern Ethiopia | 2011 | *Malaria Journal* | There is no intervention, and the outcomes were not measurement |
| 16 | Schunk et al. | High prevalence of drug-resistance mutations in *Plasmodium falciparum* and *Plasmodium vivax* in southern Ethiopia | 2006 | *Malaria Journal* | No intervention and outcomes measurement. Only genes responsible for drug resistance were investigated |
| 17 | [Schwartz et al., 1999](javascript:;) | Primaquine as prophylaxis for malaria for nonimmune travelers: A comparison with mefloquine and doxycycline | 1999 | *Clinical Infectious Diseases*, | It is prophylaxis, not interventional. No patient recruitment and follow-up |
| 18 | Teklehaimanot et al., | Case Report: Primaquine Failure for Radical Cure of *Plasmodium vivax* Malaria in Gambella, Ethiopia. | 2020 | *American Journal of Tropical Medicine & Hygiene* | Case report |

**Supplementary Table 3** ROB**-2 tools for randomized and non-randomized studies on *in vivo* efficacy of anti-malarial drugs against clinical vivax malaria in Ethiopia**

| **Study ID** | **Risk of bas: Randomized/nonrandomized/ controlled/open label clinical trials (Cochrane risk of bias tool)** | | | | | | |
| --- | --- | --- | --- | --- | --- | --- | --- |
|  | **Random sequence generation (selection bias)** | **Allocation concealment**  **(selection bias)** | **Blinding of personals & participants (performance bias)** | **Blinding of outcome assessment** | **Incomplete outcome date** | **Selective reporting** | **Other** |
| Abreha et al., 2017 | Low risk | Low risk | Low risk | Low risk | High risk | Low risk | Low risk |
|  | Patients were randomly allocated using Computer-random number generated | Patient’s identification kept in sealed opaque envelopes. | No blinding, but the review authors judge that the outcome is not likely to be influenced by lack of the blinding | Treatment failure is objective outcome and its detection is unlikely to have been affected by no blinding | There was significant number of loss to follow-up, which could affect the overall outcomes | Detailed outcomes are presented | The study protocol adhered to the WHO recommended guideline |
| Assefa et al., 2015 | Not applicable | Not applicable | Low | Low | Low | Low | High |
|  | A single-arm open-label, prospective cohort trial | A single-arm open-label, prospective cohort trial | No blinding, but the outcome is not likely to be influenced by lack of the blinding | Treatment failure is objective outcome and its detection is unlikely to have been affected by no blinding | Low dropout rate | Expected outcomes are presented | Uncorrected cure rate |
| Beyene et al., 2016 | Not applicable | Not applicable | Low | Low | Low | Low | High |
|  | A single-arm open-label, prospective cohort trial | A single-arm open-label, prospective cohort trial | No blinding, but the outcome is not likely to be influenced by lack of the blinding | Treatment failure is objective outcome and its detection is unlikely to have been affected by no blinding | Low dropout rate | Detailed outcomes are presented | Uncorrected cure rate |
| Getachew et al., 2015 | Not applicable | Not applicable | Low | Low | High | Low | High |
|  | A single-arm open-label, prospective cohort trial | A single-arm open-label, prospective cohort trial | No blinding, but the outcome is not likely to be influenced by lack of the blinding | Treatment failure is objective outcome and its detection is unlikely to have been affected by no blinding | There was significant number of loss to follow, which might affect the overall outcome | Detailed outcomes are presented | Uncorrected cure rate |
| Hwang et al. 2013 | Low risk | Low risk | Low risk | Low risk | High risk | Low risk | Low risk |
|  | Patients were randomly allocated using Computer-random number generated | Patient’s identification kept in sealed opaque envelopes. | No blinding, but the review authors judge that the outcome is not likely to be influenced by lack of the blinding | Treatment failure is objective outcome and its detection is unlikely to have been affected by no blinding | There was significant number of loss to follow, which might affect the overall outcome | Detailed outcomes are presented | The study protocol adhered to the WHO recommended guideline |
| Kanche et al., 2016 | Not applicable | Not applicable | Low | Low | Low | Low | High |
|  | A single-arm open-label, prospective cohort trial | A single-arm open-label, prospective cohort trial | No blinding, but the outcome is not likely to be influenced by lack of the blinding | Treatment failure is objective outcome and its detection is unlikely to have been affected by no blinding | Small dropout or loss to follow-up/insignificant rate | Detailed outcomes are presented | Detailed treatment responses were missing, |
| Ketema et al., 2009 | Not applicable | Not applicable | Low | Low | Low | Low | Low |
|  | A single arm open-label prospective cohort trial | A single arm open-label prospective cohort trial | No blinding, but the outcome is not likely to be influenced by lack of the blinding | Treatment failure is objective outcome and its detection is unlikely to have been affected by no blinding | Small dropout or loss to follow-up/insignificant rate | Detailed outcomes are presented | The study protocol adhered to the WHO recommended guideline |
| Ketema et al., 2011 | Not applicable | Not applicable | Low | Low | Low | Low | High |
|  | A single-arm open-label, prospective cohort trial | A single-arm open-label, prospective cohort trial | No blinding, but the outcome is not likely to be influenced by lack of the blinding | Treatment failure is objective outcome and its detection is unlikely to have been affected by no blinding | Small dropout or loss to follow-up/insignificant rate | All expected outcomes are presented | Treatment failure were not checked if they were due to true drug resistance |
| Teka et al., 2008 | Not applicable | Not applicable | Low | Low | Low | High | Low |
|  | A single-arm open-label, prospective cohort trial | A single-arm open-label, prospective cohort trial | No blinding, but the outcome is not likely to be influenced by lack of the blinding | Treatment failure is objective outcome and its detection is unlikely to have been affected by no blinding | Small dropout or loss to follow-up/insignificant rate | Important outcomes are not well presented | The study protocol adhered to the WHO recommended guideline |
| Seifu et al., 2017 | Not applicable | Not applicable | Low | Low | Low | Low | High |
|  | A single-arm open-label, prospective cohort trial | A single-arm open-label, prospective cohort trial | No blinding, but the outcome is not likely to be influenced by lack of the blinding | Treatment failure is objective outcome and its detection is unlikely to have been affected by no blinding | Small dropout or loss to follow-up/insignificant rate | All expected outcomes are presented | Uncorrected cure rate |
| Shumbej et al., 2019 | Not applicable | Not applicable | Low | Low | Low | Low | High |
|  | A single-arm open-label, prospective cohort trial | A single-arm open-label, prospective cohort trial | No blinding, but the outcome is not likely to be influenced by lack of the blinding | Treatment failure is objective outcome and its detection is unlikely to have been affected by no blinding | Small dropout or loss to follow-up/insignificant rate | All expected outcomes are presented | Uncorrected cure rate |
| Yeshanew et al., 2021 | Not applicable | Not applicable | Low | Low | Low | High | High |
|  | A single-arm open-label, prospective cohort trial | A single-arm open-label, prospective cohort trial | No blinding, but the outcome is not likely to be influenced by lack of the blinding | Treatment failure is objective outcome and its detection is unlikely to have been affected by no blinding | Small dropout or loss to follow-up/insignificant | Important outcomes are missing or unclear data presentation | Detailed treatment responses were missing |
| Yeshiwondim et al., 2010 | High risk | Low risk | Low risk | Low risk | Low risk | Low risk | Low risk |
|  | No randomization, patients were arbitrarily grouped into AL and CQ | Patients were randomly assigned to using even or odd code numbers. | No blinding, but the outcome is not likely to be influenced by lack of the blinding | Treatment failure is objective outcome and its detection is unlikely to have been affected by no blinding | Small dropout or loss to follow-up which was insignificant | All expected outcomes are presented | The study protocol adhered to the WHO recommended guideline |
| Yohannes et al., 2011 | High risk | High risk | Low risk | Low risk | Low risk | High risk | Low risk |
|  | No randomization, patients were arbitrarily grouped into AL and CQ | Patient’s identification was not kept anonymous during the grouping | No blinding, but the outcome is not likely to be influenced by lack of the blinding | Treatment failure is objective outcome and its detection is unlikely to have been affected by no blinding | Small dropout or loss to follow-up, which was insignificant | Other important outcomes not described well, e.g. PCR confirmed cases | The study protocol adhered to the WHO recommended guideline |
